# Design and Fitting Performance of a 3D-Printed Personalized Upper-Limb Socket: A Comparative Case Report

**DOI:** 10.3390/s26175374

**Published:** 2026-08-25

**Authors:** J. Carlos Díaz-Fernández, Alba Roda-Sales, Carlos Gonell-Cruz, Immaculada Llop-Harillo

**Affiliations:** Department of Mechanical Engineering and Construction, Universitat Jaume I, 12071 Castelló de la Plana, Spain; judiaz@uji.es (J.C.D.-F.); rodaa@uji.es (A.R.-S.); cgonell@uji.es (C.G.-C.)

**Keywords:** additive manufacturing, design, experimental validation, force sensors, personalized prosthetics, thermal patterns, upper-limb socket fitting

## Abstract

The prosthetic socket is the essential interface between the residual limb and the prosthesis. However, traditional upper-limb sockets are highly sensitive to residual-limb volume fluctuations and rely on experience-dependent manual fabrication processes. Additive manufacturing and 3D scanning technologies offer the possibility of producing personalized sockets, potentially improving accessibility, repeatability, and cost-effectiveness. Despite this potential, there is still a lack of systematic experimental validation directly comparing the fitting performance of personalized 3D-printed sockets with that of conventionally manufactured sockets. This study presents the design, fabrication, and comparative validation of a personalized 3D-printed upper-limb socket and a traditionally manufactured socket produced for the same user. The 3D-printed socket was developed from a 3D scanning of a clinically rectified plaster mold, computer-aided design, and material extrusion by thermal reaction bonding. A comparative validation framework evaluated both sockets under equivalent conditions, including mechanical testing, thermal behavior assessment, and sensorized fitting measurements. The results provide quantitative evidence on how a personalized 3D-printed socket performs relative to a traditional socket in terms of structural behavior, thermal patterns, and interface-related fitting parameters. This work contributes objective data supporting 3D-printed personalized sockets as a technically feasible and promising alternative to conventional methods.

## 1. Introduction

Upper-limb socket design remains one of the most critical factors in prosthetic acceptance [1]. Among all prosthetic components, the socket plays a key role, as it constitutes the physical interface between the residual limb and the prosthesis and directly determines comfort, load transfer, stability, and functional control. Inadequate socket fitting has been consistently identified as one of the leading causes of discomfort, skin injuries, reduced prosthetic control, and eventual prosthesis abandonment [2,3]. Achieving a comfortable and functional fit is particularly challenging in transradial prosthetics, where anatomical suspension relies on precise geometry above the humeral epicondyles. These fitting challenges are further aggravated by physiological changes in residual-limb volume and shape, which occur due to growth in pediatric users, weight fluctuations, muscle activity, and daily variability in adult users.

Traditionally, the sockets are fabricated through manual plaster casting, iterative hand rectifications, and resin lamination. Even though its manufacture is a highly manual and experience-dependent process, this traditional workflow represents an established baseline. Once rectified, the process is difficult to reproduce, and further adjustments must be performed again if pain or fit complaints arise. Due to the associated time and economic costs, users frequently continue wearing suboptimally fitted sockets, exacerbating the risk of tissue damage and discomfort. It is also worth noting that during resin lamination, the technicians are exposed to volatile organic compounds and toxic fumes during epoxy or acrylic curing, while presenting high time and labor costs. In addition, conventional socket fabrication requires access to specialized clinical facilities, limiting user autonomy and accessibility. Access to effective prosthetic solutions remains limited for a large proportion of the population due to the high costs associated with design, fabrication, fitting, and maintenance, particularly in low-resource settings [4].

In recent years, additive manufacturing has emerged as a promising approach to reduce costs and improve accessibility to upper-limb prosthetic devices [5]. The increased availability of low-cost 3D printing technologies, together with the rise of the “Do It Yourself” movement, has enabled rapid fabrication and customization of prosthetic components [5,6,7,8,9]. While 3D printing significantly reduces chemical exposure in the workshop environment, transitioning from conventional lamination to 3D-printed sockets requires overcoming critical design and fitting challenges. To address fitting issues, several adjustable socket concepts have been proposed, often based on modular structures using straps or tightening systems such as BOA^®^ mechanisms [10,11]. While these designs enhance adaptability, they frequently lack adequate mechanisms to control compression levels, increasing the risk of dermatological injury or insufficient fixation. Consequently, socket design remains one of the least mature and most critical limitations of 3D-printed upper-limb prostheses.

The integration of 3D scanning technologies with digital design tools and additive manufacturing workflows offers a pathway toward personalized socket fabrication [12,13]. 3D scanning enables accurate and reproducible design of sockets that closely match the desired geometry. Recent studies have begun to explore this approach, reporting improvements in fit and comfort compared to non-personalized solutions [13]. Nevertheless, the majority of existing research focuses primarily on design feasibility, manufacturing workflows, or qualitative user feedback [10,11,12].

Custom-contoured 3D design derived from a clinically rectified geometry remains a key technical strategy to ensure both proper supracondylar suspension and controlled interface pressures. However, there is a notable lack of systematic and quantitative validation studies directly comparing the performance of personalized 3D-printed transradial sockets with traditional laminated baselines under equivalent conditions. Objective evaluation of socket performance—encompassing mechanical behavior, thermal patterns, and fitting-related comfort [14]—is essential to determine whether additively manufactured sockets can reliably match or surpass conventional solutions. Objective assessment of socket fit has increasingly relied on interface pressure measurements to provide quantitative information on load transfer between the residual limb and the socket. Previous studies have demonstrated that pressure mapping can identify localized loading regions and support socket design optimization, although pressure distributions are highly subject-specific due to anatomical differences, socket geometry, and prosthetic alignment. Daly et al. [15] reported that the relationship between interface pressure and discomfort varies considerably between users, highlighting the need to interpret pressure measurements within the context of each individual rather than relying on universal pressure thresholds. Similarly, Schofield et al. [16] demonstrated that transhumeral pressure patterns differed markedly across participants and reflected individual socket design characteristics. More recent developments have incorporated embedded pressure sensors into sockets to facilitate objective socket evaluation and clinician-guided fitting [17]. Despite these advances, the limited number of upper-limb pressure studies encompassing both quantitative and qualitative aspects, and the large inter-subject variability continue to motivate investigations exploring objective methods for socket assessment. The absence of such comparative evidence remains a key barrier to the broader clinical adoption of 3D-printed upper-limb sockets, despite their potential advantages in cost, reproducibility, accessibility, and potential for digital re-fabrication without repeating the full manual process [13].

Beyond upper-limb applications, additive manufacturing has been increasingly adopted in other prosthetic and orthotic fields facing comparable challenges, such as the lack of a systematic manufacturing framework [18] or a personalized fit. In lower-limb prosthetics, additive manufacturing has been applied for socket fabrication following digital design workflows, with clinical comparisons reporting improved comfort [19,20] and reduced interface pressure relative to conventionally designed interfaces [20]. Similarly, in maxillofacial rehabilitation, additively manufactured facial prostheses have enabled highly personalized anatomical and esthetic fit for patients with craniofacial defects [21], addressing the challenges of geometric accuracy and fabrication reproducibility analogous to those encountered in socket design. These examples indicate that the technical challenges of translating personalized digital design into functional, well-fitting devices are not exclusive to upper-limb prosthetics, reinforcing the relevance of systematic and quantitative validation frameworks.

Therefore, the objective of this study is to experimentally validate the fitting of a personalized upper-limb prosthetic socket fabricated using a 3D technologies workflow derived from a clinically rectified plaster cast, and to compare its performance against a traditionally manufactured socket produced by an experienced prosthetic company. The core contribution of this work lies in the comprehensive comparative validation framework, which evaluates both socket types through mechanical and thermal testing, as well as fitting- and comfort-related assessments conducted under comparable usage conditions. By placing experimental validation at the center of the analysis, this study aims to provide a proof-of-concept evaluation regarding the technical viability of personalized 3D-printed sockets as an alternative to traditional upper-limb prosthetic sockets.

## 2. Materials and Methods

### 2.1. Socket Design and Manufacturing

Two personalized upper-limb prosthetic sockets were designed and manufactured using both 3D printing and traditional fabrication methods. A Muenster-type transradial socket was selected due to its widespread clinical use in below-elbow amputations and its ability to provide mechanical suspension through anatomical contouring above the condyles.

#### 2.1.1. Clinical Casting, Mold Rectification and 3D Scanning

**Figure 1:**
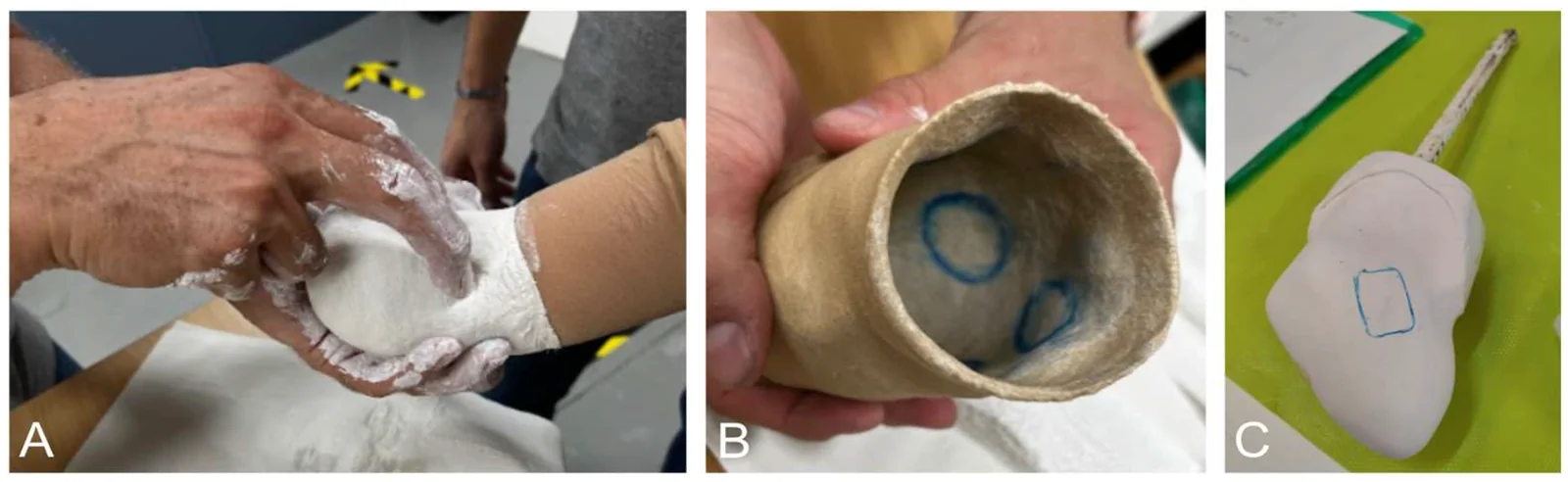
(**A**): Casting process. (**B**): Negative mold. (**C**): Rectified positive mold of the residual limb.

The residual limb of the user was initially captured using a traditional plaster casting technique (Figure 1A) by an experienced ortho-prosthetic specialist from Centro Ortopédico Sanitario (Valencia, Spain). Manual rectifications were applied by the ortho-prosthetic specialist to the negative mold (Figure 1B) to ensure adequate suspension and prevent socket slippage following clinical standards. These rectifications included localized volume reductions and pressure increases in specific anatomical regions, which are essential for achieving mechanical fixation in Muenster-type sockets. The rectified negative mold was subsequently filled with plaster to obtain a positive mold of the residual limb (Figure 1C). This positive plaster mold (Figure 1C) was used as the reference geometry for both sockets: that obtained through traditional fabrication methods and that obtained through a digital workflow and 3D-printed. This approach was selected not only to ensure that both sockets were based on the same clinically validated residual-limb geometry, thereby improving experimental reproducibility, but also because the residual limb contains deformable soft tissues whose geometry may change substantially depending on the applied external pressure and loading conditions. Direct scanning of the residual limb may capture a geometry that differs from the functional shape established through clinical rectification and subsequently adopted within the socket interface.

For obtaining a personalized 3D-printed socket, the use of a clinically rectified plaster cast represents a hybrid workflow that combines conventional clinical practice with digital design and additive manufacturing. The potential relevance of this workflow lies in combining patient-specific fabrication with a low-cost and reproducible additive manufacturing process, which remains a relevant challenge in the field of upper-limb prosthetic socket development.

#### 2.1.2. Geometric Modeling

**Figure 2:**
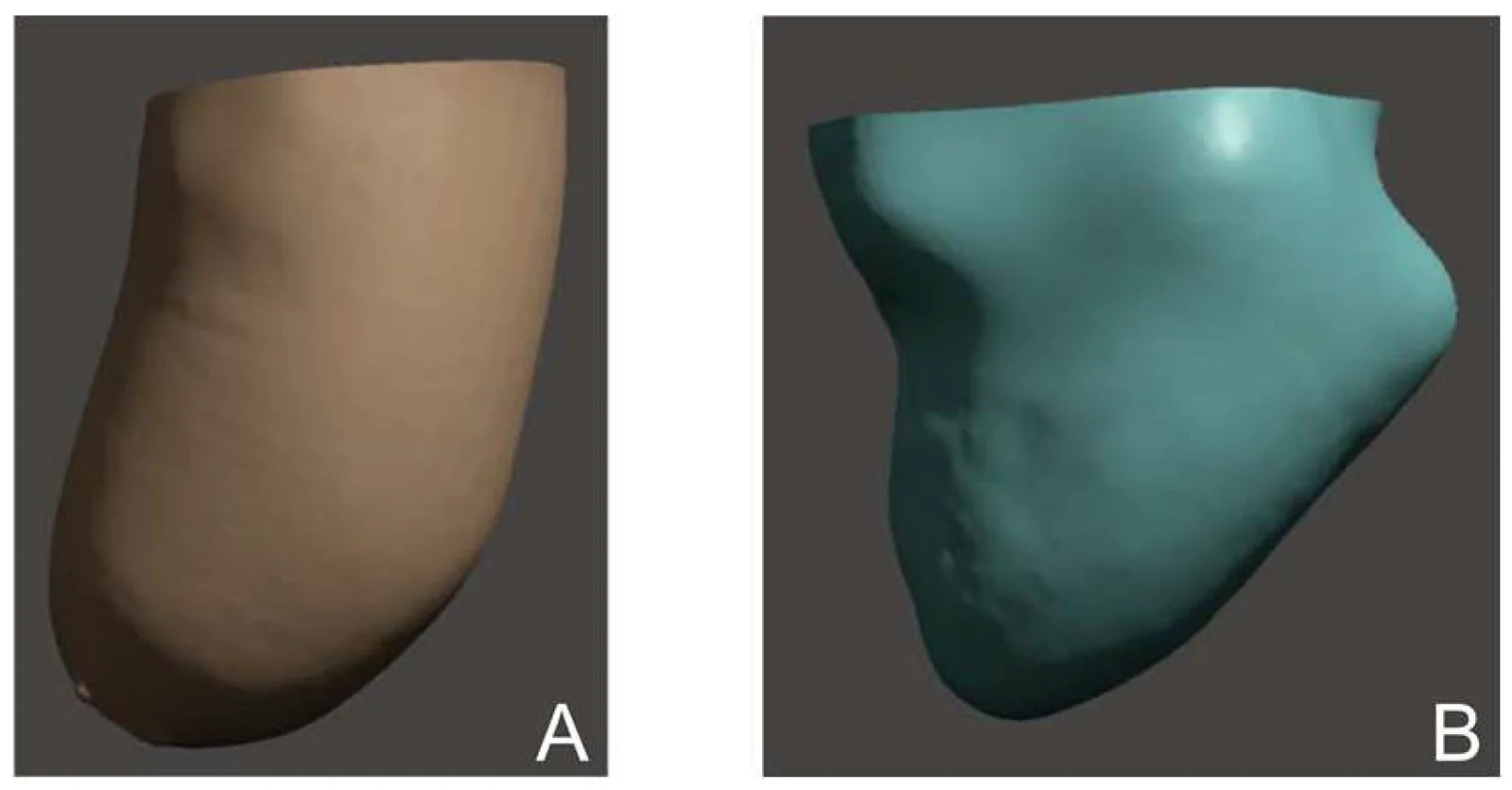
(**A**): 3D scan of the residual limb. (**B**): 3D scan of the rectified positive mold.

The positive plaster mold (Figure 1C) was digitized using the Sense^TM^ 3D Scanner from 3D Systems, Inc. (Rock Hill, SC, USA) (Spatial X/Y resolution at 0.5 m: 0.9 mm; depth resolution at 0.5 m: 1 mm), generating a triangulated surface mesh (STL format) of the rectified residual-limb geometry (Figure 2B). This STL model constituted the geometric basis for the design of the 3D-printed prosthetic socket and the remaining customized components.

Figure 2 shows the 3D scan of the residual limb (A) and the 3D scan of the positive rectified mold (B) to illustrate the principal morphological modifications introduced during the rectification process by the ortho-prosthetic specialist. The purpose of this visualization is illustrative rather than quantitative. The observed modifications include increased clearance in the biceps region to allow elbow flexion, relief zones around the epicondyles and olecranon, and a deliberate volume reduction above the olecranon to enhance mechanical suspension. Moreover, a higher below-elbow soft tissue volume is observed in the residual-limb scan compared to the mold scan.

In addition to the residual-limb geometry, anthropometric measurements of the user’s contralateral (non-affected) forearm were recorded and used as reference values to guide the design of the prosthetic forearm. These measurements were preserved as closely as possible to ensure appropriate mass distribution, avoid unnecessary weight increases that could affect prosthetic handling, and achieve a natural and symmetric esthetic appearance. Furthermore, circumferential measurements of the contralateral forearm were taken at proximal, medial, and distal regions and incorporated into the design process. These perimeters were considered not only for aesthetic consistency but also to ensure proper integration of the terminal device, which interfaces with a dedicated distal forearm component.

The rectified residual limb STL model was imported into Meshmixer (Autodesk Inc., USA, v3.5.474) for further processing and socket modeling. The complete digital workflow used to generate a 3D-printable socket geometry from the scanned model is provided in Appendix A to ensure full reproducibility.

#### 2.1.3. Additive Manufacturing and Assembly

**Figure 3:**
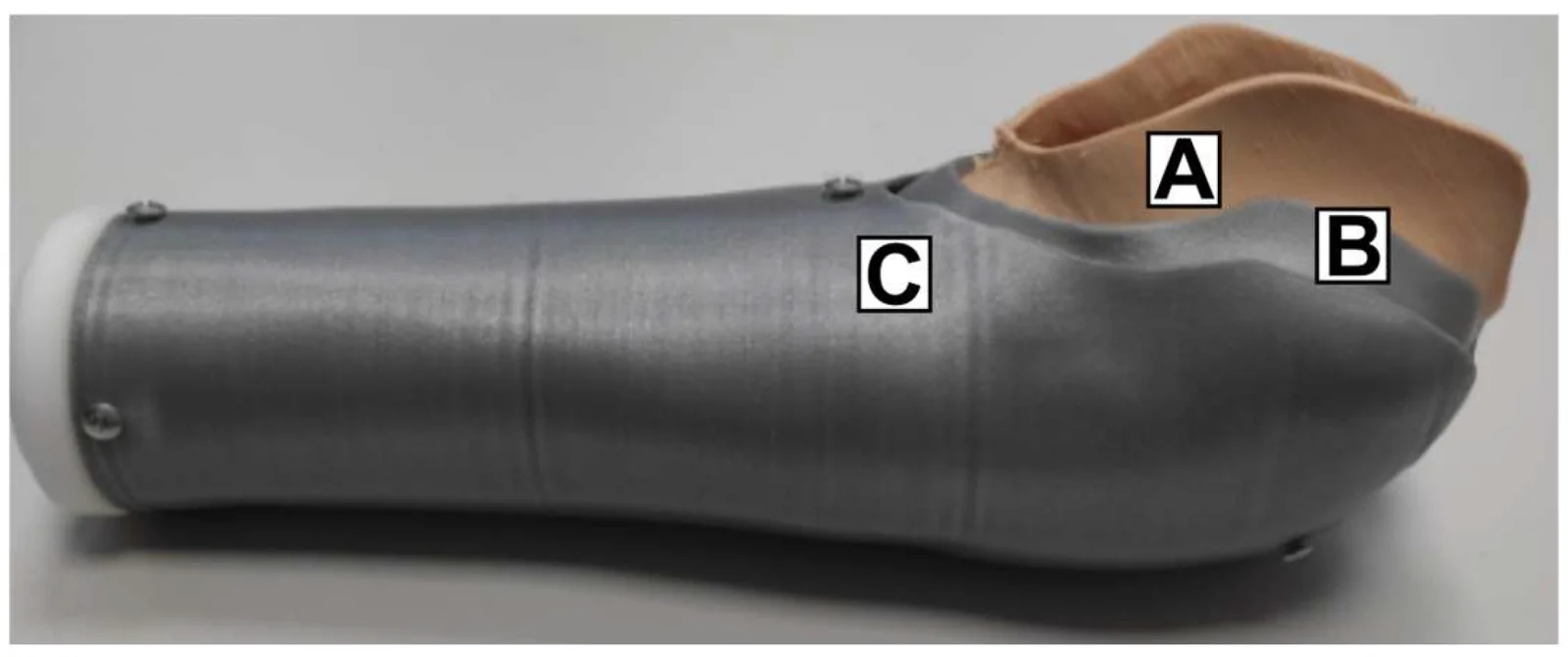
3D-printed assembly: (A) soft socket, (B) mechanical connector, (C) forearm structure.

The customized prosthetic structure consisted of three main patient-specific components: (A) the soft socket, (B) the mechanical connector, and (C) the forearm structure (Figure 3). These components were designed to be assembled together and coupled with a commercially available prosthetic wrist (model REGAL 4-02-WH; Regal Prosthesis, Hong Kong, China), which served as the interface for the terminal device used during experimental testing.

All customized components were fabricated using material extrusion by thermal reaction bonding (MEX-TRB [22]). Different materials were selected according to the functional requirements of each component.

The soft socket was printed using a flexible thermoplastic polyurethane (TPU, Filaflex, Shore 82A), selected for its combination of high elasticity, excellent elastic recovery, and high tear resistance, which are desirable characteristics for prosthetic socket applications requiring conformity to the residual limb while maintaining structural integrity. Furthermore, the material has been developed for flexible additive manufacturing applications and has undergone biocompatibility testing according to ISO 10993 [23,24,25], making it suitable for prolonged skin contact.

For the connector and forearm structure, polylactic acid (PLA) was selected due to its compatibility with low-cost, unenclosed desktop material extrusion printers, low warping, absence of hazardous fume emission during processing, and low material cost, in line with the accessibility objectives of this study. However, PLA is known to be sensitive to time- and temperature-dependent changes in mechanical behavior [26]. Within the scope of the present short-term feasibility study, these effects are not expected to influence the results. Materials such as ABS, ASA, nylon, or fiber-reinforced PLA composites may offer improved long-term durability but at the expense of higher material cost and more demanding printing requirements.

The 3D printing parameters used for the fabrication of each component are summarized in Table 1.

The soft socket component exhibited minor filament stringing, a known artifact associated with flexible filaments and low printing speeds. In contrast, the rigid components (connector and forearm) presented a smoother surface finish. Dimensional distortions were observed at the wrist interface of the forearm component, requiring post-processing prior to assembly performed by mechanical removal of material in regions of interference to enable proper insertion of the commercial wrist component.

Threaded M3 heat-set inserts were embedded into the connector using a thermal insertion tool to ensure secure mechanical fastening. The wrist was fixed to the forearm using four self-tapping M3 screws. Subsequently, the connector was attached to the forearm using three M3 screws. The soft socket was inserted into the connector by elastic deformation and press-fitting, achieving secure fixation once the deformation forces were released. The final assembly is shown in Figure 3.

#### 2.1.4. Traditionally Manufactured Socket

As a baseline for the experimental comparison of this work, a traditionally manufactured socket was fabricated by EMO company (Especialidades Médico Ortopédicas SL, Valencia, Spain). The fabrication process was based on the positive plaster mold of the participant’s residual limb previously obtained (Figure 1C).

**Figure 4:**
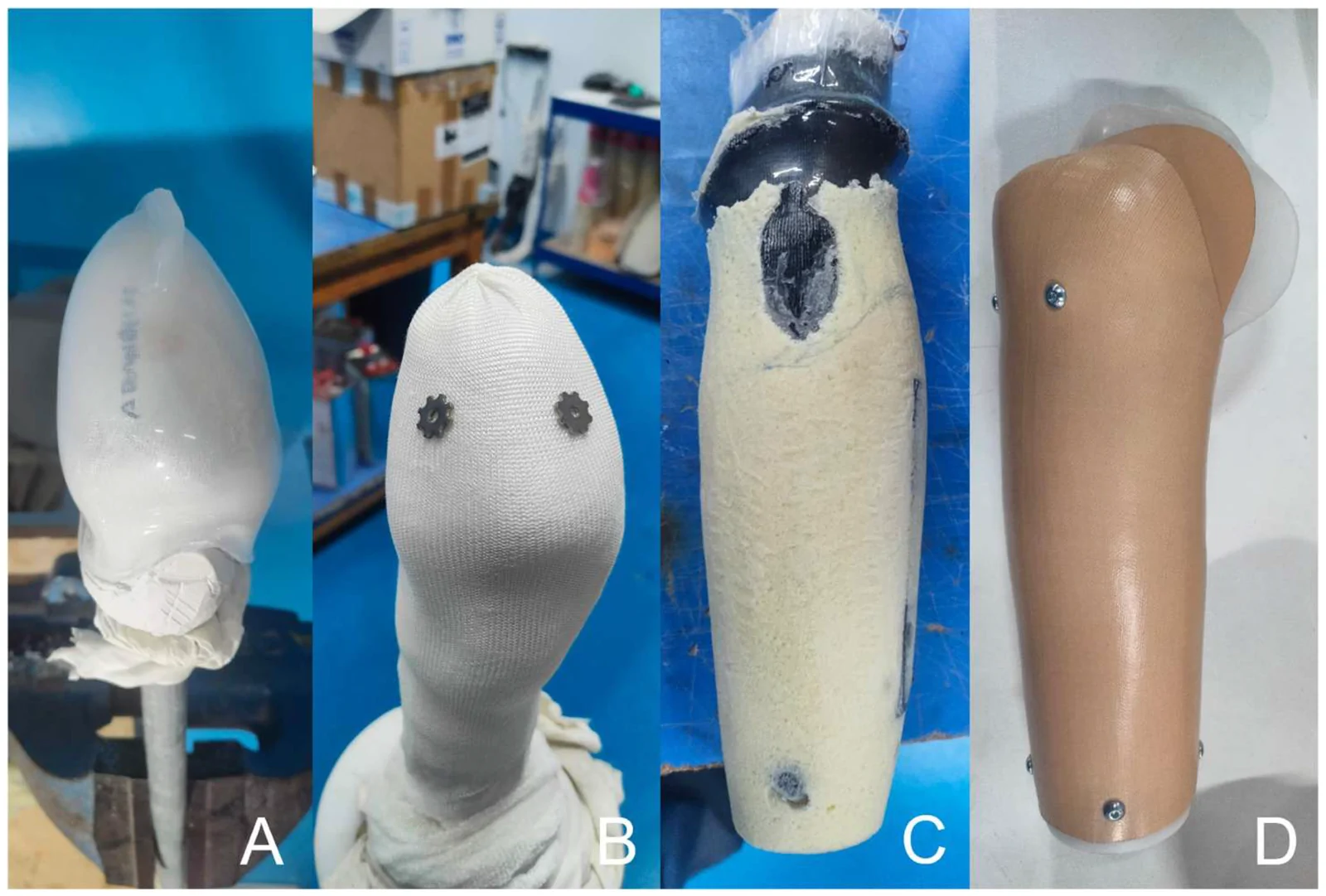
Traditional socket manufacturing: (**A**) soft socket molding, (**B**) connector preparation for lamination, (**C**) forearm structure conformation, (**D**) final assembly.

First, the soft socket component was thermoformed using extruded ethylene–vinyl acetate (EVA) directly over the positive mold of the residual limb (Figure 4A). Subsequently, a fabric liner was applied over the EVA component (Figure 4B), and the elbow connector was fabricated through a lamination process using glass and nylon fiber (ny-glass) composites impregnated with acrylic resin.

The forearm structure was then manufactured by modeling a polyurethane foam core according to the anatomical measurements of the participant’s forearm (Figure 4C). This structure was subsequently laminated using the same glass and nylon fiber composite with acrylic resin to obtain the final rigid forearm component.

Finally, once all socket components were fabricated, the wrist unit (Regal Prosthesis; REGAL 4-02-WH), identical to the one used in the 3D-printed socket, was integrated, and the complete assembly was finalized (Figure 4D).

### 2.2. Socket Sensorization

**Figure 5:**
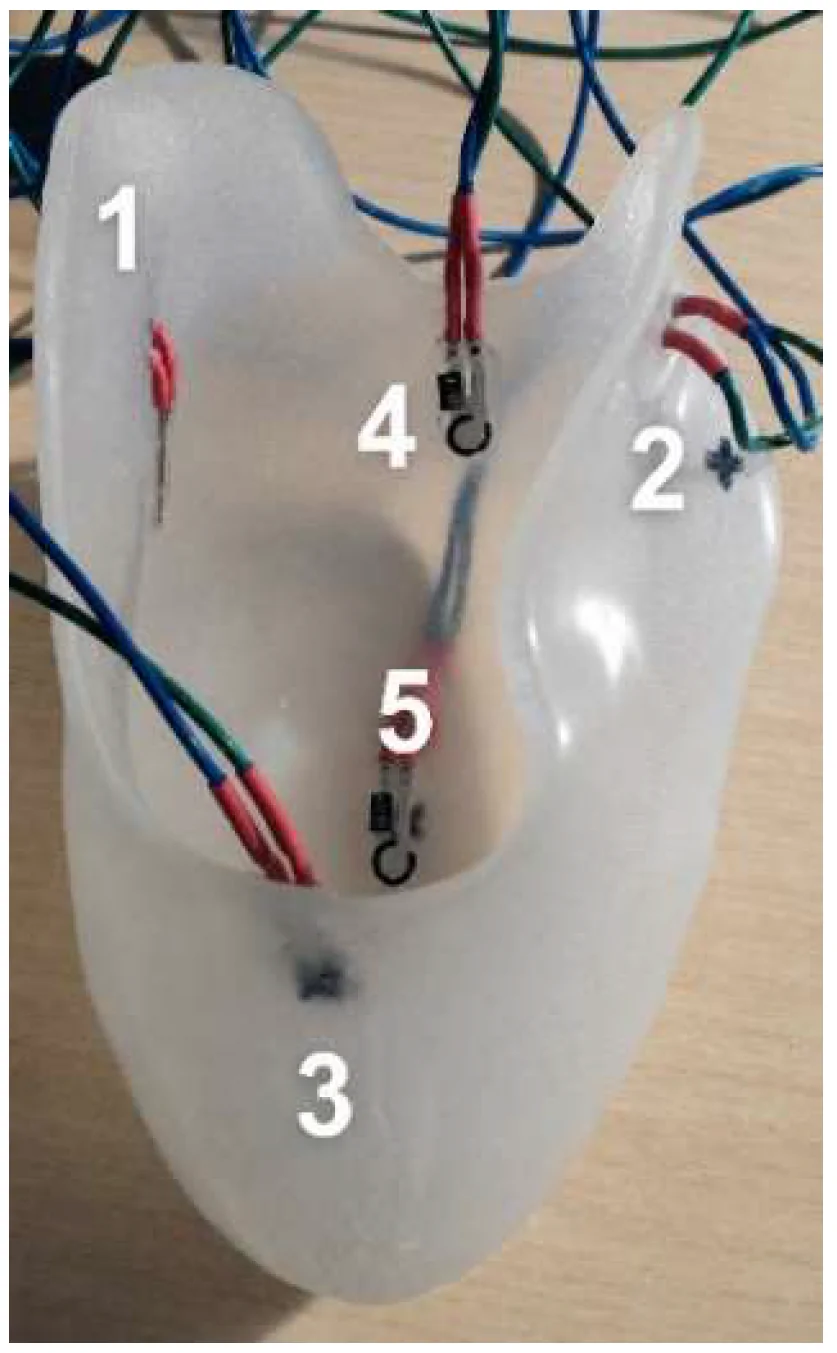
Sensors location used in both types of sockets: (1) right epicondyle, (2) left epicondyle, (3) anterior soft tissue, (4) olecranon, (5) ulna.

To quantitatively compare the interface forces acting on the residual limb during testing the 3D-printed socket and the traditionally manufactured socket, force sensors were integrated into each socket structure. Five thin-film force sensors (FlexiForce A101, Tekscan Inc., Norwood, MA, USA) were positioned at anatomically relevant regions associated with socket suspension and load transfer around the elbow. These locations correspond to areas where mechanical anchoring of the Muenster-type socket occurs, including zones near the epicondyles and olecranon, where pressure concentration is expected. The specific locations, used in both types of sockets, are detailed in Figure 5. The integration of the sensors was achieved by making precise perforations at the specific areas, so the active sensing area of the sensors remained in direct contact with the participant’s limb, while the wiring was routed externally to avoid any discomfort or interference with the fit of the socket. To ensure consistent positioning between trials, the sensors were fixed using double-sided medical tape.

The Tekscan FlexiForce A101 sensors operate using force-sensitive resistor (FSR) technology. When a normal force is applied to the sensing area, the conductive polymer layer reduces its resistance, producing a measurable voltage change. Their reduced thickness (≈0.203 mm) and flexibility make them suitable for integration inside the socket without significantly altering the fit or user comfort. However, given that these sensors do not directly output force values but rather a resistance variation dependent on the applied load, a custom electronic interface and signal conditioning strategy were required. The system was designed to convert mechanical loads occurring at the stump–socket interface into quantifiable digital signals using an Arduino-based acquisition platform. Particular attention was paid to portability, mechanical robustness, and participant safety, as the measurements were performed during dynamic trials involving human interaction. Moreover, these sensors require application-specific calibration, as their measurement range and sensitivity depend on the electrical conditioning circuit.

#### Measurement System Architecture

The sensorized system consisted of the prosthetic socket, five Tekscan FlexiForce A101 force sensors, five 100 kΩ reference resistors, an Arduino Uno microcontroller board (Arduino, Monza, Italy), a prototyping board, connection wiring and a USB communication cable, and a computer for data acquisition and storage.

Each sensor was connected in a voltage divider configuration in order to convert its force-dependent resistance variation into a measurable voltage signal (sampling frequency: 20 Hz). Each of the five sensors were connected in series with a fixed reference resistor (100 kΩ ±5%), forming a voltage divider supplied by the Arduino 5 V output. The Arduino acquired the output voltage (*V*_0_) (measured at the junction between the sensor and the reference resistor) from each sensor through its analog input channels. The Arduino Uno features a 10-bit analog-to-digital converter, providing 1024 discrete levels over the reference voltage range. The analog value measured by the sensor is read using the Arduino function ‘analogRead()’ and, once obtained, was converted to voltage. Then, based on the voltage divider configuration and Ohm’s law, the sensor resistance was derived and subsequently related to the applied force through the calibration curve, which was obtained experimentally after a calibration procedure. The calibration is essential to establish a precise mathematical transfer function that maps the raw digital units back to physical force units, such as kilograms or Newtons.

The calibration process accounts for the inherent non-linear characteristics of thin-film force sensors. The FlexiForce A101 typically exhibits a power–law relationship between the applied load and its electrical response. By performing a systematic characterization across a known range of loads, it is possible to derive a regression equation that compensates for this non-linearity. Calibration was performed using a digital dynamometer with known load measurement accuracy of ±0.01 kg. A rigid metallic tip was used to apply compressive force centrally over the active sensing area in order to ensure repeatable loading conditions. Loads were applied incrementally from 0 to 1 kg in 0.1 kg intervals, as this was the range of loads expected during socket usage. At each load step, the force was held constant for 3 s, and measurements were repeated 3 times to reduce variability. The different values of “*V*_0_” and variable resistance were recorded and saved into an Excel spreadsheet. The sensor’s behavior was obtained through two calibration curves, applied force vs. sensor resistance and applied force vs. measured voltage (*V*_0_). Regression analysis revealed that the voltage-based model provided a superior coefficient of determination (R^2^ =0.9528). Therefore, the final calibration equation implemented in the Arduino was:

(1) $$Force\;=\;0.3675\cdot V_{0}^{-1.196}\;(kg)$$

Additionally, to account for the sensors’ inherent baseline offset, a detection threshold of 61.7 g (raw analog value of 1010) was established; values below this limit were recorded as 0 g. This baseline fluctuation, primarily driven by electronic noise, required a software-based filtering approach for both the traditional and the 3D-printed sockets. This measure effectively defined the system’s minimum detectable force, ensuring that only genuine mechanical interactions were recorded and preventing false-positive pressure readings during the experimental trials.

According to the manufacturer, the sensors can measure forces up to approximately 44 N; however, increasing the measurement range by modifying the circuit gain may reduce sensitivity and increase thermal drift. Therefore, the conditioning circuit was configured to prioritize sensitivity within the force range expected at the socket–limb interface. Typical sensor uncertainties include ±2.5% repeatability error, ±3% linearity error, and up to ±5% hysteresis, leading to a worst-case total error of approximately 10%.

**Figure 6:**
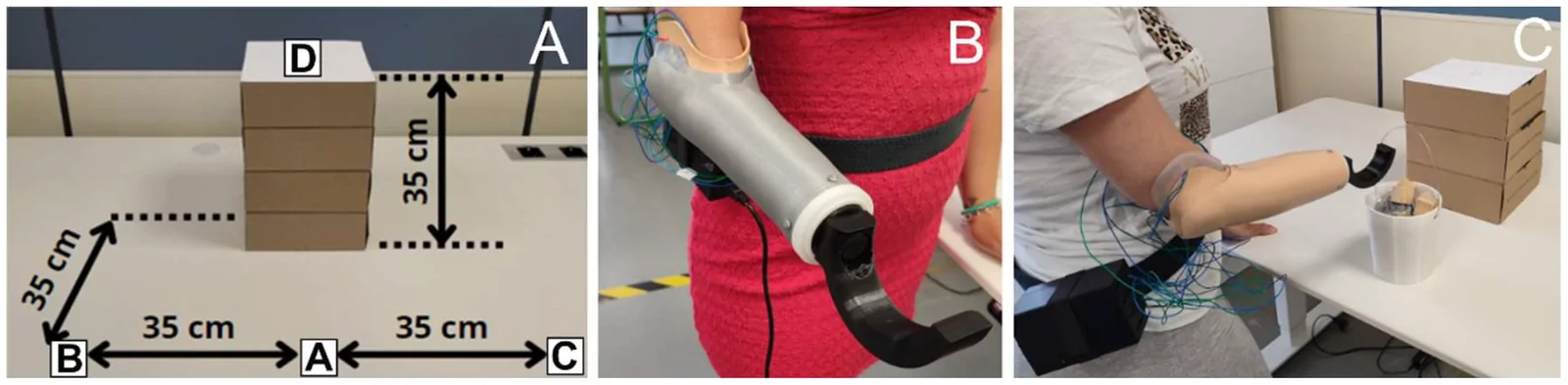
(**A**): Workspace. (**B**): Experimental setup using the 3D-printed socket. (**C**): Experimental setup using the traditionally manufactured socket.

Following the circuit design, the physical implementation was optimized for portability and robustness. The hardware was assembled on a breadboard where the Arduino was mounted underneath the board itself. This stacked configuration was chosen to create a more compact system and optimize the overall footprint of the electronics. Moreover, given that the data collection involved trials with a human participant, a custom enclosure was designed and 3D-printed to house the breadboard and Arduino assembly (Figure 6C). This housing was essential to prevent relative movement between components, protect the equipment from accidental impacts, and ensure the stability of the electrical connections during the movements inherent in the testing process. The enclosure design included specific apertures for the USB power/data cable and dedicated slots for the sensor leads to exit the box without being pinched. Furthermore, the upper section of the case featured a specialized slot for a Velcro strap, providing a secure closure mechanism. For the duration of the experimental sessions, this encapsulated system was attached to the participant’s waist using a belt (see Figure 6B,C), allowing for a portable and non-intrusive setup that ensured the participant’s comfort while maintaining the integrity of the sensor data.

### 2.3. Experiments Performed

The experimental protocol was designed to evaluate the forces experienced at the socket–residual limb interface, the thermal behavior of the residual limb and the socket, the skin condition of the residual limb, and the user’s experience after using both upper-limb prosthetic sockets during standardized pick-and-place tasks under different loading conditions. This holistic approach was adopted to integrate both objective and subjective measures. While interface force measurements provide objective information on load distribution and potential indicators of socket fitting, thermographic imaging offers complementary information on surface thermal behavior that can be interpreted alongside the force measurements. These objective assessments were further complemented by photographic documentation of the residual limb and subjective user feedback regarding comfort, sweating, thermal sensation, pain, and perceived socket stability, providing a comprehensive evaluation of socket performance.

The study involved a single participant with a congenital transradial agenesis. The participant provided informed consent prior to the experimental session.

#### 2.3.1. Experimental Setup

All measurements were conducted in a controlled laboratory environment. The workspace consisted of an adjustable-height table set at 75 cm, simulating standard table height, with designated start and end points indicated by stickers on the table, for pick-and-place tasks. Four points were defined: three on the table surface (A, B, and C) and one elevated (D), all separated by 35 cm distances (see Figure 6A).

The prosthetic sockets evaluated were the traditionally manufactured socket and the 3D-printed one. In both cases, the terminal device was a 3D-printed hook designed in SolidWorks 2024 SP5 (Dassault Systèmes, Vélizy-Villacoublay, France) and mounted onto the wrist and secured using an M12 × 1.5 screw (see Figure 6B,C). These prostheses were used to pick-and-place standardized loads of 1 kg and 1.5 kg using a loaded bucket (see Figure 6C). The bucket was designed in SolidWorks and 3D-printed with a wire handle and loads were adjusted using combinations of wooden and metallic parts to achieve the desired mass.

#### 2.3.2. Instrumentation

Force measurements were recorded using the setup described in Section 2.2. To complement the force measurements, thermographic images were acquired with a Flir i7 infrared thermal camera (FLIR Systems, Wilsonville, OR, USA) immediately after completion of the tasks to visualize the thermal behavior of the participant’s residual limb and the inner surface of the socket.

#### 2.3.3. Task Protocol

The participant performed different-object pick-and-place tasks, involving 24 combinations of weight, direction, and height of movement, with each socket during a session on different days. Each pick-and-place task required a single movement in one of eight directions: rightward, leftward, upward, downward, or diagonally (upward or downward to either side) between points (A, B, C, D, depicted in Figure 6A). Each task was performed handling two loads: firstly, the 12 tasks were carried out with a 1 kg bucket to pick and place, and later with a 1.5 kg bucket. Prior to data collection, the participant was allowed to familiarize herself with the tasks and equipment. Each trial began after the participant confirmed readiness and was verbally cued to start. Force data read by the Arduino was sent via USB cable to a laptop and was recorded continuously during task execution and saved immediately after each trial.

Following task completion, the socket was removed and additional measurements were collected, including surface thermography of the residual-limb and inner-socket surfaces, as well as photographic documentation of skin condition. Thermal images were acquired immediately after socket removal under the same environmental conditions (same laboratory and controlled temperature) and using the same camera settings, acquisition distance and anatomical views for both socket conditions to ensure comparability. Moreover, the participant completed a post-task questionnaire (see Appendix B).

### 2.4. Data Analysis

All recorded data, including force sensor outputs, thermal images, photographs, and questionnaire responses, were systematically organized and stored for subsequent analysis.

Force sensor data from the five Tekscan FlexiForce A101 sensors were processed to generate time-series plots for each task, allowing visualization of load distribution across the socket and identification of potential localized overloads during specific movements. Thermographic images were analyzed to identify surface temperature patterns within the socket and on the residual limb, providing complementary information regarding thermal behavior and differences in socket–limb interaction. Photographic documentation was reviewed to detect signs of skin irritation or pressure marks resulting from socket use. Responses from the post-task questionnaire were compiled and summarized to assess subjective perceptions of discomfort, pain, sweating, thermal sensation, and perceived stability of the prosthetic socket.

This multi-modal analysis enabled a comprehensive evaluation of both mechanical and user-centered performance of the personalized 3D-printed socket compared to the traditional socket under equivalent usage conditions and obtained from the same rectified positive mold.

## 3. Results

### 3.1. Force Sensor Measurements

**Figure 7:**
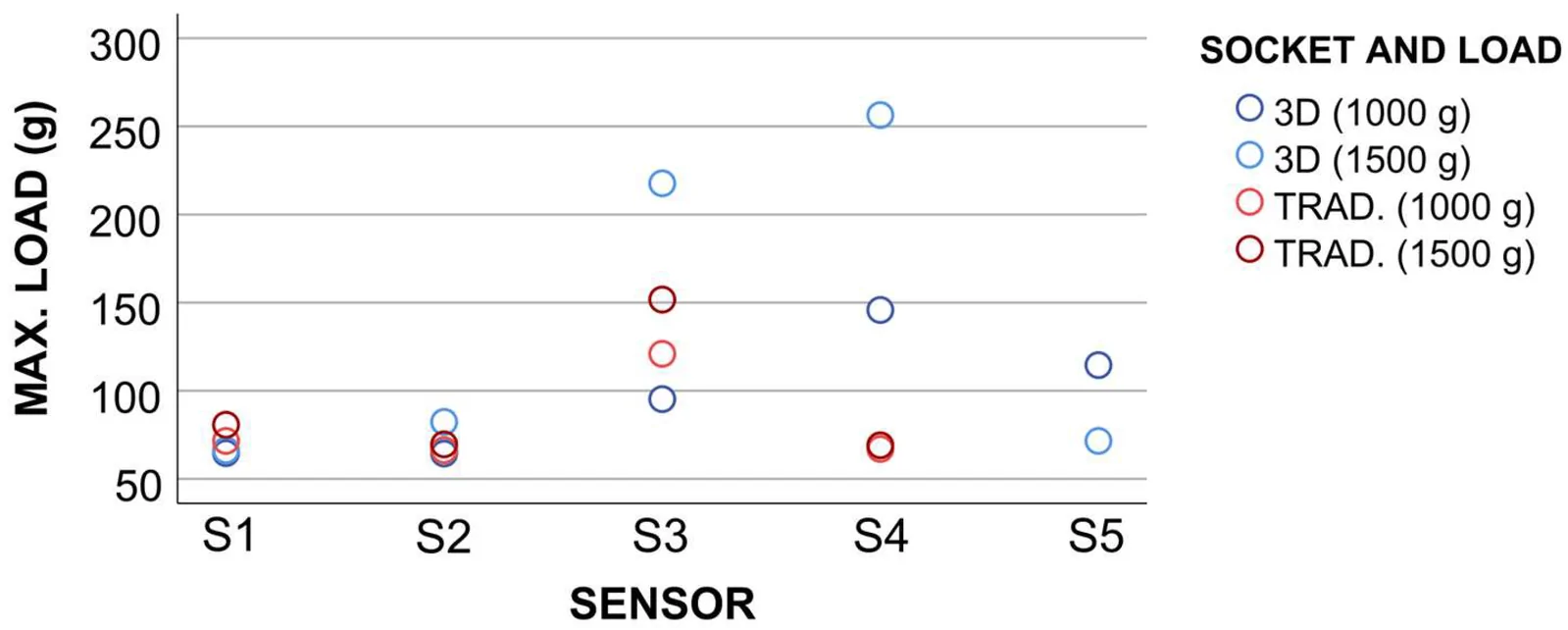
Overview of sensor maximum load recorded during pick-and-place tasks with each socket type and task load.

The plots of the force measurements recorded by the five Tekscan FlexiForce A101 sensors (see Figure 5) are presented in Appendix C and Figure 7 presents a summary of the maximum loads recorded by each sensor when using both types of sockets with both loads. The full dataset of collected force data is available as Supplementary Material.

For the traditional socket, Sensor 3, located on the anterior upper portion of the socket, registered the highest forces, with peaks of 120.9 g during the 1 kg trials and 151.7 g during the 1.5 kg trials. Considering the sensor area, the maximum pressure detected in the traditional socket was 131.22 kPa corresponding to 31.33% of the reported pain threshold of 418.8 kPa [27]. It is also worth noting that Sensor 5, situated near the proximal ulna, consistently measured 0 g throughout the trials, likely due to limited contact with the residual limb and forces below the detection threshold.

In the 3D-printed socket, Sensor 4, positioned over the olecranon, was generally the most active, peaking at 145.8 g for the 1 kg trial and 256.4 g for the 1.5 kg trial. Sensor 3, on the anterior upper portion, also registered significant forces, reaching 95.2 g and 217.2 g for the 1 kg and 1.5 kg trials, respectively. Sensors 1, 2 and 5 showed moderate variations. The maximum calculated pressure in the 3D-printed socket reached 221.78 kPa, representing 52.95% of the pain threshold [27].

**Figure 8:**
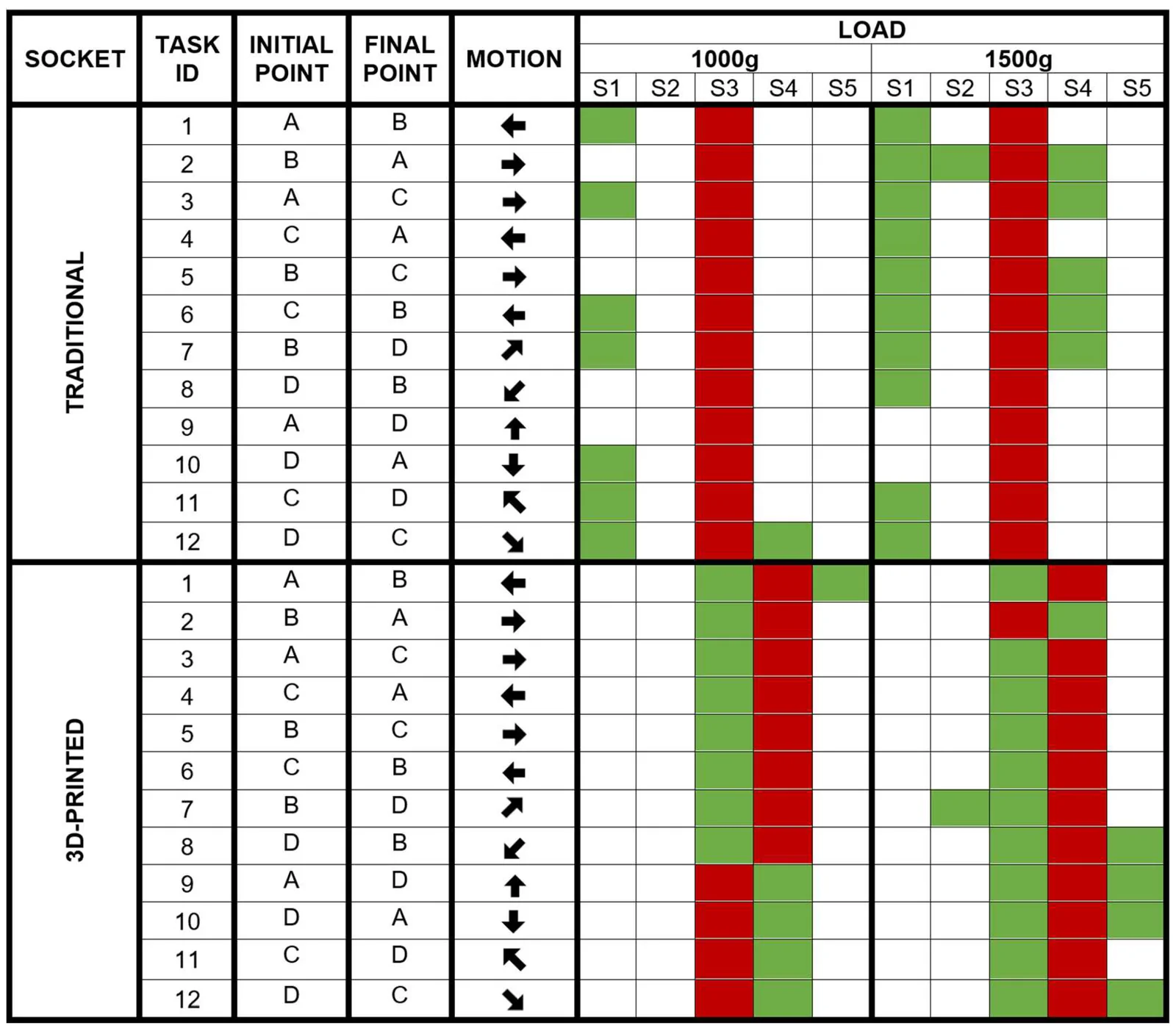
Overview of sensor activation (S1–S5) across tasks, along with the motion and the initial and final point of each task, according to the points detailed in Figure 6A. Sensors exhibiting a signal variation higher than 5 g are highlighted in color, with red indicating those that recorded the highest pressure values in the task.

Overall, both sockets maintained forces below the pain threshold, with the 3D-printed socket exhibiting higher peak pressures, particularly at the olecranon and anterior regions. Figure 8 provides an overview of sensor activation across tasks (highlighting sensors with pressure variation and pressure maximums), along with a detail of the motion performed in each task and their initial and final point in the experiment scenario, according to the points shown in Figure 6A.

### 3.2. Thermal Measurements

**Figure 9:**
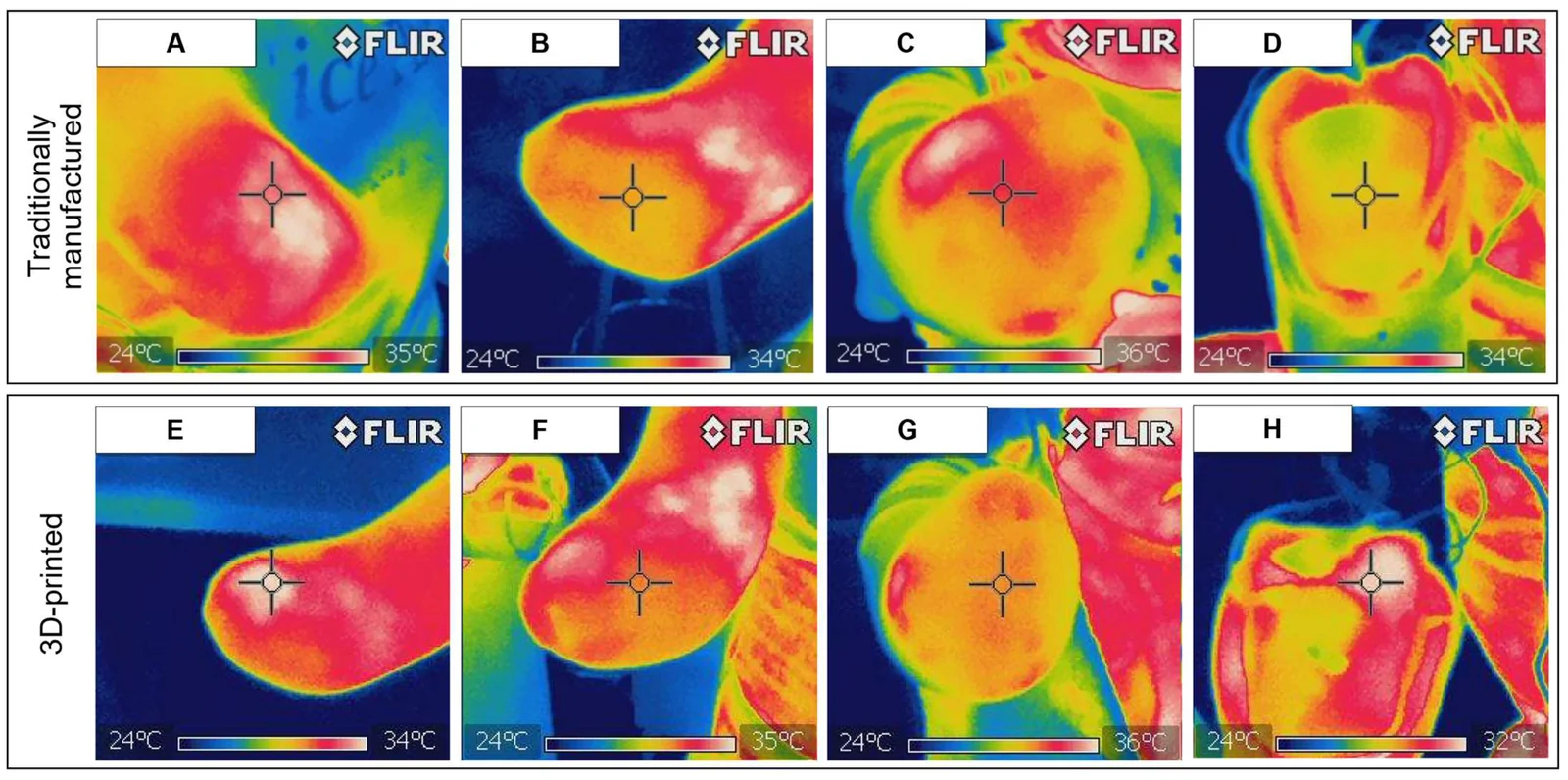
Thermal images of the residual limb (**A**–**C**) and the inner part of the socket (**D**) after using the traditionally manufactured socket and thermal images of the residual limb (**E**–**G**) and the inner part of the socket (**H**) after using the 3D-printed socket.

For the traditional socket, the highest temperatures were spatially coinciding with Sensors 3 (Figure 9A), 1 (Figure 9C), 4 and 2 (Figure 9B), areas where the highest force values were recorded. Meanwhile, the inferior region of the limb, corresponding to Sensor 5, displayed the lower temperatures and corresponded to an area where minimal force values were detected (Figure 9B). When observing the temperatures in the inner part of the socket (Figure 9D), the traditional socket showed higher temperatures concentrated in areas associated with active flexion of the forearm.

For the 3D-printed socket, thermal imaging showed also highest temperatures coinciding with Sensors 3 (Figure 9E), 4 and 2 (Figure 9F). But in general, these thermal patterns were more evenly distributed in this socket than in the traditionally manufactured one. When observing the temperature distribution on the inner surface of the socket (Figure 9H), also a uniform pattern could be observed, which may indicate homogeneous contact between the socket and the residual limb and load transfer across the interface.

Although infrared thermography does not provide direct measurements of interface pressure, it is a useful complementary technique for identifying thermal patterns associated with socket contact and potential areas of heat accumulation, even though the comparison based on these figures is limited since the areas of interest have not been isolated. Besides that, it has to be noted that the observed differences may also be influenced by the thermal properties of the materials used in the soft-socket components. The traditional socket was fabricated from EVA, which has low thermal conductivity (0.036–0.04 W/m·K), whereas the 3D-printed socket used TPU with higher thermal conductivity (0.14–0.5 W/m·K), which may contribute to differences in heat transfer during socket use.

### 3.3. Skin and Visual Inspection

**Figure 10:**
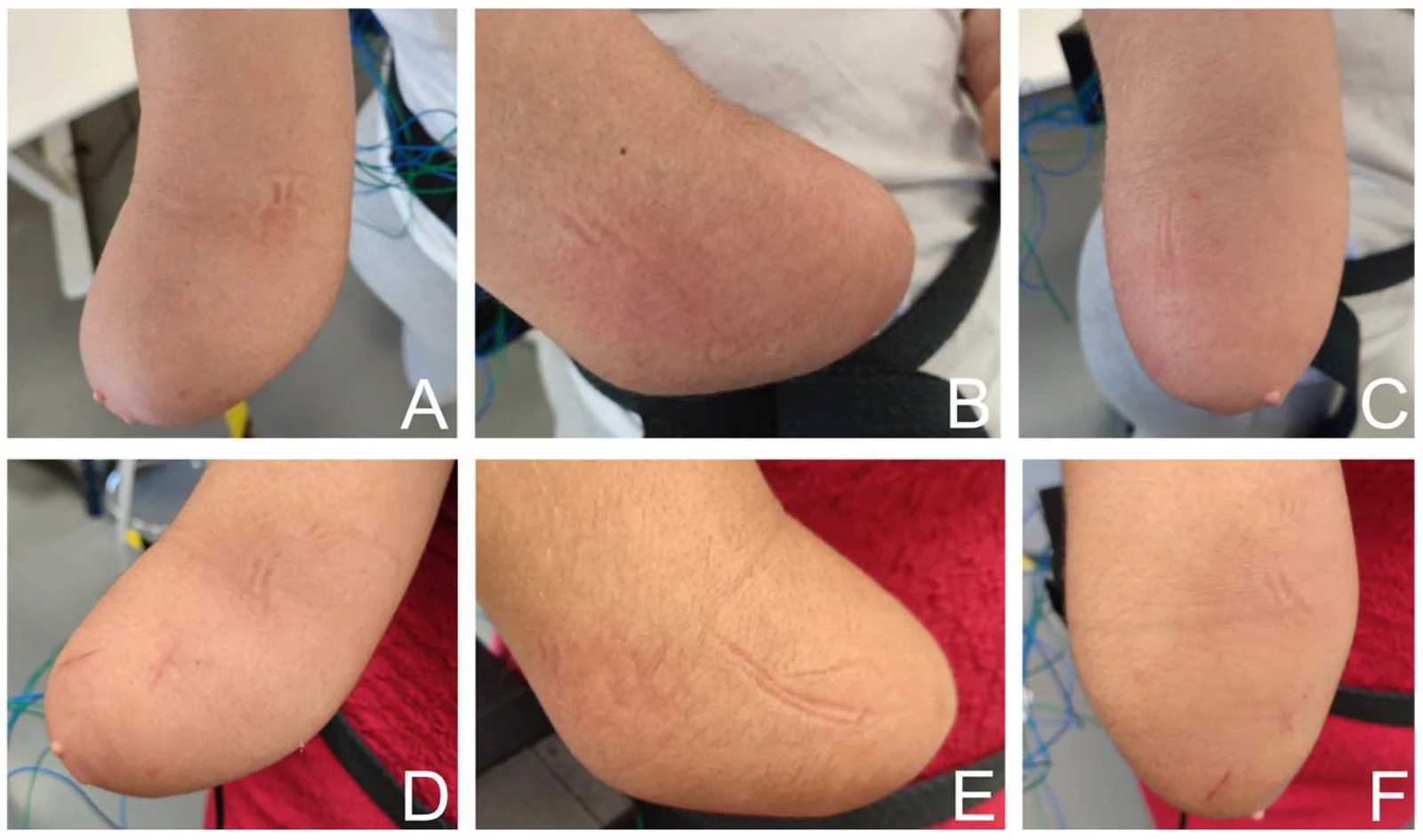
Images of the limb after using the traditionally manufactured socket (**A**–**C**) and the 3D-printed socket (**D**–**F**).

Visual inspection of the residual limb was performed immediately after the experimental trials to identify potential dermatological effects associated with socket use. Representative photographs of the inner, outer, and superior views of the limb were obtained for both sockets (Figure 10).

Neither the traditional socket (Figure 10A–C) nor the 3D-printed socket (Figure 10D–F) showed clinically relevant skin alterations (irritation, inflammation, or dermatological lesions). The skin condition remained comparable between both types of sockets. In both, mild skin imprints corresponding to the locations of the force sensors were visible, accompanied in some areas by slight transient redness. These marks were localized and consistent with the presence of external measurement devices rather than socket structure itself. There is no evidence of persistent redness, abrasions, or pressure-related injury. It should be noted that small superficial scratches are visible in some photographs (Figure 10D,F), but these were already present prior to the experiment and were not caused by the prosthetic socket or the experimental tasks.

Overall, visual assessment after the use of both sockets indicated that short-term socket use under the tested loads did not produce adverse dermatological effects on the residual limb.

### 3.4. Subjective Questionnaire Results

The participant did not report pain, discomfort, or skin irritation during the use of either socket. In both cases, she indicated a sensation of stability and security while performing the tasks. No perception of localized overheating inside the socket was reported for either design.

A difference between sockets was observed in perceived sweating. During use of the traditional EVA socket, the participant reported mild sweating, whereas no sweating sensation was reported during use of the 3D-printed TPU socket.

No qualitative suggestions for improvement were provided in the structured questionnaire for either socket. However, when asked to compare both systems after completing all trials, the participant expressed a clear preference for the 3D-printed socket. The reasons given included reduced sweating, lower rigidity compared with the EVA socket, better overall adaptation to the limb, reduced friction sensation, and improved handling due to lower weight. She also reported greater comfort in the cubital fossa region when using the 3D-printed socket, which is consistent with the lower force concentration observed experimentally in the anterior soft-tissue area when compared with traditional socket use.

## 4. Discussion

### 4.1. Force Measurements

The force measurements indicate that the traditional socket did not fully engage the distal portion of the residual limb during use. This observation is corroborated by Sensor 5 readings, located in this area, which did not activate throughout the trials. In contrast, the 3D-printed socket achieved measurable force distribution across the entire residual limb, including the distal region, suggesting improved anatomical conformity and load transfer.

Notably, in the 3D-printed socket, the highest forces were detected at the olecranon (Sensor 4), rather than in the anterior forearm region where the traditional socket exhibited peak forces. This shift in load concentration is advantageous, as the olecranon region provides a firmer bony support, while the anterior region consists mainly of soft tissue more susceptible to discomfort and skin irritation. Consequently, although the peak forces in the 3D-printed socket were higher than those in the traditional socket, these forces remained below the established pain threshold [27] and the user reported less friction and discomfort during use.

The observed redistribution of peak forces from the anterior soft tissues toward the olecranon region may be related to both geometric conformity and material behavior. The digital design workflow allowed precise control of the socket’s internal geometry and local anatomical contours, which may have resulted in closer conformity around the olecranon and consequently facilitated greater load transfer to this bony region. At the same time, the lower stiffness and viscoelastic behavior of TPU may have enabled greater local compliance and adaptation of the socket to the residual limb during loading, potentially reducing localized pressure over the softer anterior tissues. These factors may have acted synergistically to produce the more balanced load distribution observed in the 3D-printed socket. However, because geometry and material properties were not independently varied in the present study, further investigations are required to determine the relative contribution of each factor.

Overall, these preliminary findings demonstrate that personalized 3D-printed sockets can maintain forces within safe limits while providing subjective comfort compared to traditionally manufactured sockets, supporting their preliminary technical feasibility as an alternative workflow. Nonetheless, pressure measurements should not be considered a replacement for clinical evaluation or user-reported outcomes, but rather an objective complement that provides quantitative information on socket–limb mechanical interaction before secondary effects, such as discomfort or skin irritation, become clinically apparent.

It should be noted, however, that one limitation of this study concerns the potential influence of the MEX-TRB manufacturing process on the final geometry of the 3D-printed socket. Layer-by-layer deposition involves repeated heating and cooling cycles that may generate residual thermal stresses and dimensional deviations, potentially causing differences between the nominal digital geometry and the 3D-printed socket. Such deviations could affect socket fit and load distribution independently of the intended digital design. Although the present study demonstrated differences in biomechanical loading between the two socket types, dimensional deviations between the digital model and the final printed socket were not independently quantified. Therefore, the contribution of manufacturing-induced geometric changes cannot be excluded. Future studies should incorporate dimensional verification of the 3D-printed socket, for example, through post-print 3D scanning and comparison with the original digital model, to distinguish the effects of intended geometric design from those introduced during additive manufacturing.

### 4.2. Thermal Measurements

The thermographic results provide complementary information to the force sensor measurements by revealing differences in thermal patterns associated with socket–limb interaction. The combination of both approaches suggests that the 3D-printed socket enabled a more balanced contact distribution across the residual limb. In the traditional socket, areas of increased temperature were observed in regions where higher forces were also recorded, particularly in the frontal superior area. This spatial correspondence may indicate greater contact and mechanical interaction in these regions, which may be associated with discomfort during prolonged use. Conversely, the 3D-printed socket facilitated effective contact in previously unloaded regions, such as the inferior portion of the residual limb, reducing the concentration of heat in the flexion region and distributing it more evenly.

The improved thermal behavior of the 3D-printed socket can be attributed both to its material properties and the personalized fit obtained through 3D scanning. TPU’s higher thermal conductivity compared to EVA may have contributed to differences in heat transfer behavior between both socket designs. However, thermal patterns are also influenced by contact conditions, wearing time, environmental factors, and skin characteristics. Additionally, the personalized socket design ensured proper limb–socket coupling across all regions, allowing pressures to be distributed over more thermally conductive areas, such as the olecranon, rather than localized soft-tissue regions prone to irritation.

### 4.3. Skin Aspect

The absence of significant skin irritation or inflammatory signs in both socket types suggests that the pressure levels recorded during the trials were well tolerated by the residual-limb tissues. The superficial marks observed were primarily associated with the placement of force sensors rather than with the socket–limb interface itself. These marks are therefore considered an artifact of the measurement setup and not representative of everyday prosthetic use.

Importantly, despite differences in force distribution patterns between the traditional and 3D-printed sockets, no negative dermatological outcomes were observed with either design. This finding supports the interpretation that, although the 3D-printed socket redistributed loads toward the olecranon region and produced higher peak forces, these remained within physiologically tolerable limits. The lack of visible skin compromise reinforces the safety of the pressure levels measured and aligns with the thermographic and sensor data indicating absence of harmful overloading.

Taken together, the visual inspection results complement the mechanical and thermal findings, indicating that both sockets were safe under the tested conditions, while the 3D-printed socket achieved improved load distribution without increasing the risk of skin irritation.

### 4.4. Subjective Perception and Its Correlation with Objective Data

The subjective feedback provided by the participant is consistent with the mechanical and thermal findings obtained during the experimental trials. Although both sockets were reported as stable and free of pain, relevant differences emerged in perceived sweating, comfort, and overall preference, which can be directly related to the measured interface conditions.

The absence of pain for both sockets is coherent with the calculated interface pressures, which remained below the reported pain threshold [27]. Even in the most unfavorable case (3D-printed socket), peak pressures did not reach levels associated with tissue pain, supporting the participant’s report of pain-free use.

The difference in perceived sweating between sockets was consistent with the thermographic observations and may be influenced by differences in material properties. The lower thermal conductivity of EVA compared with TPU may affect heat transfer at the skin–socket interface and could contribute to differences in perceived thermal conditions. However, thermal and moisture behavior within the socket environment is influenced by multiple factors beyond material composition, including socket geometry, wall thickness, contact conditions, wearing time, and surface characteristics derived from the manufacturing process. Although the 3D-printed socket presented a higher wall thickness than the traditional socket (2.34 mm vs. 2.25 mm), lower sweating perception was reported. This suggests that the interaction between the skin and socket surface, rather than thickness or material properties alone, may influence the local microclimate. The layered structure inherent to additive manufacturing may modify the surface characteristics and local contact conditions, potentially affecting moisture exchange at the skin–socket interface; however, further studies measuring humidity, airflow, and surface roughness would be required to confirm this hypothesis.

Regarding perceived comfort, the reported improvement experienced at the cubital fossa when using the 3D-printed socket is particularly relevant. Force sensor measurements showed that, in the traditional socket, the anterior soft-tissue region (associated with forearm flexion) experienced the highest load concentrations, whereas in the 3D-printed design, peak forces were shifted toward the olecranon region, which is anatomically better suited to tolerate mechanical loading. This redistribution of forces from compliant soft tissue to more load-tolerant anatomical structures provides a plausible biomechanical explanation for the reduced friction sensation and improved comfort reported by the participant.

The preference for the 3D-printed socket due to lower rigidity and improved adaptation to limb morphology is also coherent with the observed contact behavior. The traditional socket showed incomplete contact in the distal region, while the 3D-printed socket achieved measurable load transfer in this area, indicating a more uniform interface. A better fit between socket and limb likely reduces localized pressure peaks and micromovements, factors commonly associated with discomfort and skin irritation.

Finally, the perception of improved handling due to lower weight highlights an additional functional advantage of additive manufacturing approaches, which allow weight reduction without compromising structural integrity. The weight of the traditional prosthetic assembly was 297.5 g, whereas the weight of the 3D-printed socket was 276.5 g. This represents a 7.06% reduction in the overall assembly weight compared to the traditional prosthetic assembly, which may influence long-term user acceptance and prosthesis wear time.

Overall, the subjective responses reinforce the objective findings and support the conclusion that the 3D-printed socket provides improved biomechanical load distribution and a slightly enhanced thermal and functional comfort, even when absolute pressure values are slightly higher than those measured with the traditional socket.

## 5. Conclusions

This study assesses the biomechanical and thermal performance of a traditionally manufactured upper-limb prosthetic socket and a socket obtained using 3D technologies (3D scanning and printing), both of which were made from the same rectified positive mold. The results indicate that both sockets provide safe interface conditions, with pressure levels remaining below reported pain thresholds and no clinically relevant skin alterations observed. Subjective feedback further confirmed adequate comfort, stability, and absence of pain in both configurations.

The 3D-printed socket demonstrated more evenly distributed loads relative to the traditional socket, including effective contact in the distal region, and a shift in peak loads toward anatomically more load-tolerant areas such as the olecranon. Thermal observations and user perception suggested slightly improved heat distribution and reduced sweating when using the TPU-based printed socket. These differences were associated with enhanced comfort perception, particularly in regions of soft tissue.

Importantly, the findings do not indicate a superiority that invalidates conventional sockets. Instead, is shown that when a digital workflow is based on proper clinical rectification of a plaster cast and subsequent 3D scanning, additively manufactured sockets can achieve a level of fit and biomechanical performance comparable to traditionally fabricated ones. Additive manufacturing further introduces practical advantages such as design flexibility, weight reduction, reproducibility, and potentially lower production costs, which may improve accessibility to customized prosthetic solutions.

Additionally, future developments could incorporate topology optimization tools driven by Finite Element Analysis (FEA) in the forearm structure. This approach would allow for targeted material removal in low-stress regions, further reducing total weight while ensuring structural integrity and customized fit.

Overall, this feasibility study provides initial evidence that 3D-printed sockets, when derived from a rectified clinical cast, can achieve fitting performance comparable to traditional sockets, being an accessible alternative to conventional fabrication. Nevertheless, this work has been conducted on a single subject under controlled short-term conditions. Consequently, the observed pressure profiles and thermal behavior cannot be directly extrapolated to the broader amputee population. Further work regarding long-term clinical viability or dermatological safety requires further validation through longitudinal clinical trials involving larger populations, extended wear durations, and real-world functional tasks.

## Figures and Tables

**Table 1:** 3D printing parameters of the 3D-printed socket components (soft socket, mechanical connector and forearm structure).

3D Printing Parameter	Soft Socket	Mechanical Connector	Forearm Structure
Placement	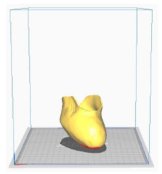	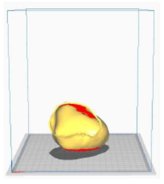	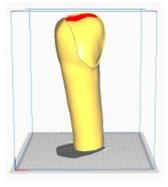
Printer	Ender 3 S1 Pro	Prusa MK4S	Ender 3 S1 Pro
Material	TPU 82A	PLA	PLA
Nozzle Temperature (°C)	230	210	210
Bed Temperature (°C)	80	60	60
Print Speed (mm/s)	20	80	60
Wall Thickness (mm)	0.2	0.2	0.2
Infill Density (%)	40	40	40
Infill Pattern	Zig Zag	Zig Zag	Zig Zag
Supports	Yes (Organic/Tree)	Yes (Organic/Tree)	None
Build Plate Adhesion	Skirt	Skirt	Skirt

**Figure A1:**
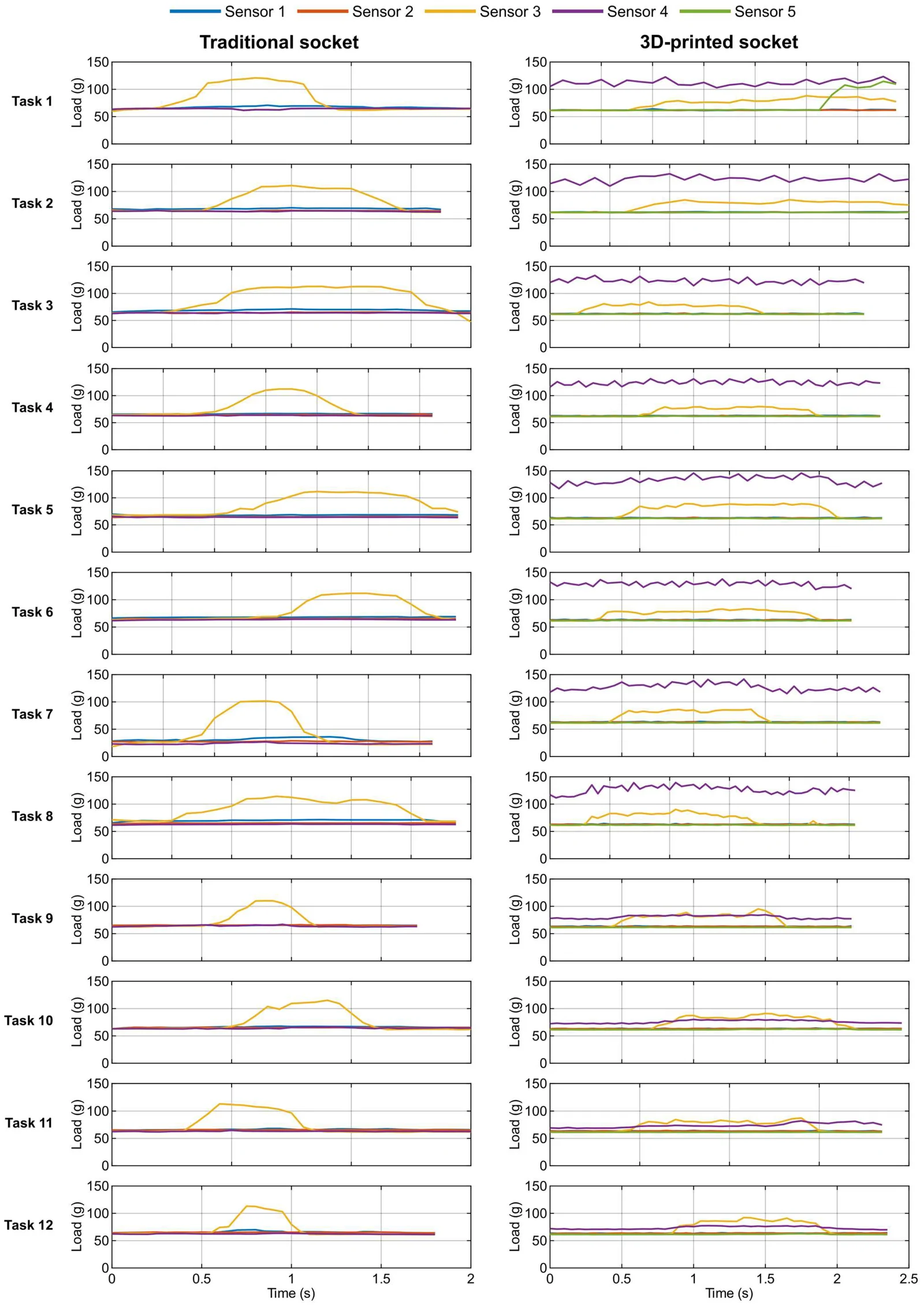
Time series of force sensor data for each socket and task performed under a 1000 g load.

**Figure A2:**
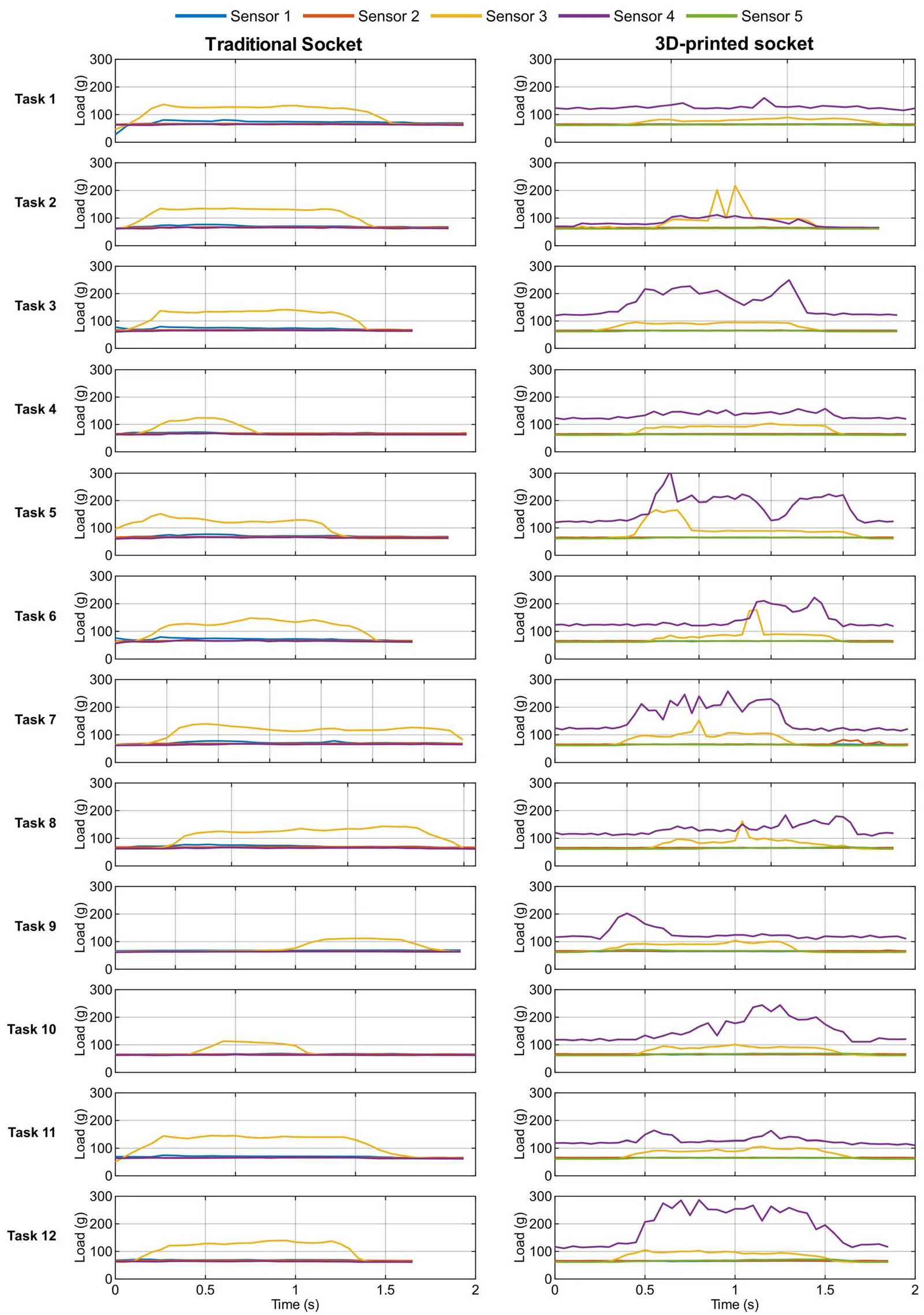
Time series of force sensor data for each socket and task performed under a 1500 g load.

## Data Availability

The original contributions presented in this study are included in the article/Appendix A, Appendix B and Appendix C/Supplementary Material. Further inquiries can be directed to the corresponding author.

## Supplementary Materials

The following supporting information can be downloaded at: https://www.mdpi.com/article/10.3390/s26175374/s1. File S1: 3DSocket_ForceData.xlsx; File S2: TraditionalSocket_ForceData.xlsx.

## Abbreviations

The following abbreviations are used in this manuscript:

3D	Three-dimensional
EVA	Ethylene–vinyl acetate
FEA	Finite element analysis
MEX	Material extrusion
PLA	Polylactic acid
TPU	Thermoplastic polyurethane
TRB	Thermal reaction bonding

## Appendix A. Socket Modeling Process

This appendix provides a detailed description (Table A1, Table A2 and Table A3) of the modeling of each part of the 3D-printed socket using Meshmixer (Autodesk Inc., San Francisco, CA, USA, v3.5.474).

### Appendix A.1. Soft Socket

**Table A1:** Soft-socket modeling process.

ID	Step	Parameters	Figure
1	Importing the .STL file corresponding to the scan of the rectified positive mold of the residual limb. Contouring of the socket shape over the 3D model of the residual limb using the “Select” command.	Size: 55	
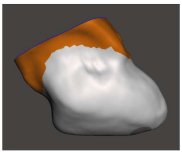
2	Smoothing of the contour using the “Smooth Boundary” command under the “Modify” menu	Smoothness: 90 Preserve Shape: 90 Iterations: 90 Border Rings: 3	
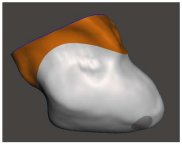
3	Removal of regions not belonging to the socket using the “Discard” tool under the “Edit” menu.	None	
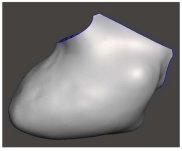
4	Rounding of edges and surface smoothing using the “Sculpt” tool.	Brushes: Robust Smooth Strength: 25 Size: 50 Brushes: Flatten Strength: 15 Size: 20	
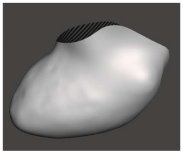
5	Extruding the “soft socket” contour using the “Offset” tool in “Edit”.	Distance: 2 mm Accuracy: 90 Regularity: 90 Soft Transition: 0 mm Connected	
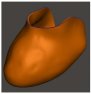
6	Verification of mesh quality using the “Wireframe” command under the “View” menu.	None	
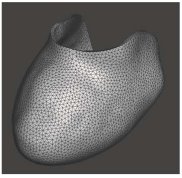

### Appendix A.2. Soft Socket

**Table A2:** Connector modeling process.

ID	Step	Parameters	Figure
1	Importing the “soft socket” part. Contouring of the connector contour over the soft socket part using the “Select” tool.	Brush Mode: Unwrap Brush Size: 55 Expand Mode: Geodesic Distance Not Allow Back Faces	
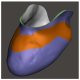
2	Selection of the entire connector surface using the “Select” tool.	Size: 90	
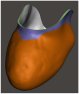
3	Smoothing the edges using the “Smooth Boundary” tool in “Modify”.	Smoothness: 90 Preserve Shape: 90 Iterations: 95 Border Rings: 2	
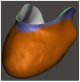
4	Separating the connector from the socket by 0.5 mm on each side using the “Extract” tool in “Edit”.	Direction: Normal	
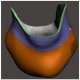
5	Mesh correction using the “Sculpt” tools and checking the mesh quality with the “Show Wireframe” command in “View”.	Brushes: Robust Smooth Strength: 30 Size: 30	
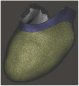
6	Extruding the connector using the “Offset” tool in “Edit”.	Distance: 2 mm Accuracy: 60 Regularity: 60 Soft Transition: 0 mm Connected	
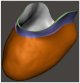

### Appendix A.3. Forearm

**Table A3:** Forearm modeling process.

ID	Step	Parameters	Figure
1	Importing the “connector” part. Outlining the contour of the forearm over the “connector” part using the “Select” tool.	Brush Mode: Unwrap Brush Size: 55 Expand Mode: Geodesic Distance	
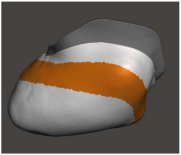
2	Selecting the entire surface of the forearm’s organic part using the “Select” tool.	Size: 90	
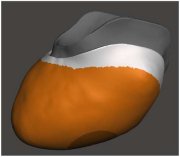
3	Smoothing the contour using the “Smooth Boundary” tool in “Modify”.	Smoothness: 90 Preserve Shape: 90 Iterations: 95 Border Rings: 2	
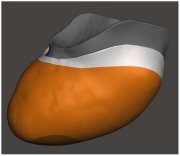
4	Separating the organic part of the forearm from the connector by 0.5 mm on each side using the “Extract” tool in “Edit”.	Direction: Normal	
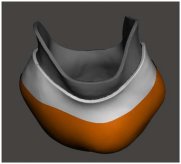
5	Smoothing the mesh using the “Sculpt” tools and checking the mesh quality with the “Show Wireframe” command in “View”.	Brushes: Robust Smooth Strength: 15 Size: 30	
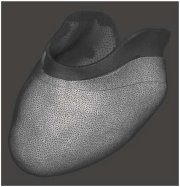
6	Extruding the organic part of the forearm using the “Offset” tool in “Edit”.	Distance: 2 mm Accuracy: 60 Regularity: 60 Soft Transition: 0 mm Connected	
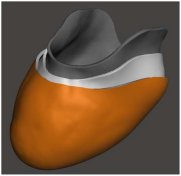
7	Creating the end of the forearm (wrist) in SolidWorks.	Diameter: 54.5 mm Extrusion: 5 mm	
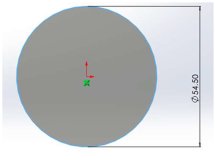
8	Importing the “wrist” part into Meshmixer and aligning it with the organic part of the forearm.	None	
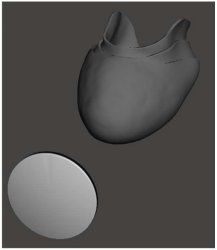
9	Creating a cutting plane. Removing the area defined by the plane using the “Discard” tool in “Edit”.	Cut type: Slice (Keep Both) Fill Type: No Fill	
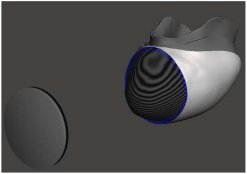
10	Removing imperfections using “Close Cracks” in “Edit”. Removing the inner cap of the wrist using the “Discard” command in “Edit”.	None	
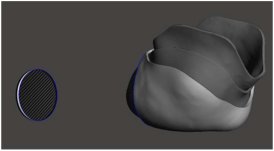
11	Joining the edge of the wrist with the forearm using the “Join” command.	None	
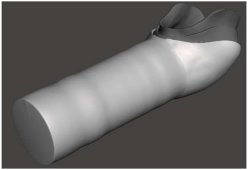
12	Smoothing the joint using the “Smooth” command in “Deform” and the “Sculpt” command.	None	
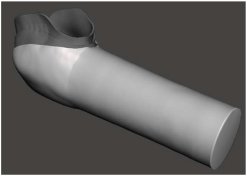
13	Hollowing the forearm part using the “Hollow” command.	Offset Distance: 2 mm Solid Accuracy: 256 Mesh Density: 128	
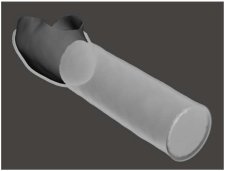
14	Creating an opening in the wrist using a cutting plane 225 mm from the olecranon.	None	
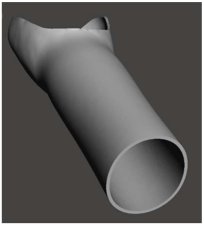
15	Smoothing the inside of the forearm to achieve the final geometry using the “Sculpt” command.	Brushes: Robust Smooth Strength: 20 Size: 30	
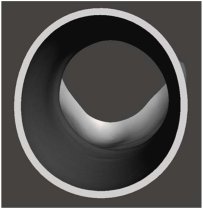

## Appendix B. Questionnaire

Upon completion of all tasks using both prosthetic sockets, the participant was asked to complete a questionnaire to evaluate aspects such as comfort, breathability, and other relevant factors. The list of questions and their corresponding response formats (e.g., 1–10 rating scale or yes/no) are presented in Table A4.

**Table A4:** Questions asked to the participant after the experiment.

ID	Question	Response Type
1	Did you experience pain while using the prosthesis?	Yes/No
2	If yes, indicate where and intensity	Likert 1–10
3	Did you notice discomfort while using the prosthesis?	Yes/No
4	If yes, indicate where and intensity	Likert 1–10
5	Did you notice higher temperatures in the socket area?	Yes/No
6	If yes, indicate where.	Open answer
7	Did you experience sweating?	Yes/No
8	If yes, indicate level.	Mild/Moderate/Severe
9	Did you experience skin irritation?	Yes/No
10	If yes, indicate areas and severity	Likert 1–10
11	Did the socket provide stability and safety?	Yes/No
12	If no, rate instability.	Likert 1–10
13	Comments or suggestions for improvement.	Open answer

## Appendix C. Force Sensor Data

This appendix presents the complete time-series data acquired from the five force sensors embedded within the sockets during the experimental trials. The full dataset is available as Supplementary Material. The purpose of this section is to provide a detailed visualization of the force distribution patterns across all tasks and loading conditions, complementing the summarized results discussed in the main manuscript.

Two sets of experiments were conducted under different loading conditions (1000 g and 1500 g). For each condition, force signals recorded from all five sensors are shown for every task, allowing a direct comparison between the traditional socket and the 3D-printed socket.

Figure A1 and Figure A2 display the temporal evolution of the measured loads for each sensor across the twelve tasks performed. Each subplot corresponds to a specific task, while different colors represent individual sensors. This representation enables the identification of peak loads, load distribution trends, and differences in sensor activation between socket designs.

These plots serve as a qualitative and quantitative reference to support the analysis of variations in load concentration, stability, and contact behavior between both socket configurations.
